# Creative Collaboration and Collaborative Creativity: A Systematic Literature Review

**DOI:** 10.3389/fpsyg.2021.713445

**Published:** 2021-08-09

**Authors:** Margaret S. Barrett, Andrea Creech, Katie Zhukov

**Affiliations:** ^1^Sir Zelman Cowen School of Music and Performance, Monash University, Melbourne, VIC, Australia; ^2^Schulich School of Music, McGill University, Montreal, QC, Canada

**Keywords:** creative collaboration, collaborative creativity, collaborative creative learning, distributed creativity, cultural psychology, music performance, improvisation, composition

## Abstract

Studies of creativity emerging from cultural psychology and social psychology perspectives challenge individualist conceptions of creativity to argue that social interaction, communication, and collaboration are key elements in creativity. In recent work creative collaboration has been proposed to be “distributed” between audiences, materials, embodied actions, and the historico-socio-cultural affordances of the creative activity and environment, thus expanding the potentialities of creative collaboration beyond instances of direct human interaction and engagement. Music performance, improvisation and composition may be viewed as exemplary “laboratories” of creative collaboration through the combined elements of audiences, materials, embodied actions and historico-socio-cultural affordances and constraints. This article reports the findings of a systematic literature review of creative collaboration and collaborative creativity in music. We sought to identify what has been currently investigated in relation to these terms and concepts in music, with what methodologies and in what settings. Findings indicate that studies were undertaken in higher education, professional development and professional practice predominantly, leading to an emergent phenomenon of interest, collaborative creative learning. Musical genres were jazz, popular, western classical, contemporary and world musics across the musical processes of composing, improvising and performing. Studies in higher education and professional development settings focused on identifying those practices that supported learning rather than the nature of collaborative creative approaches or the outcomes of creative collaboration. Participants were primarily male, with small sample sizes. Methodologies were largely qualitative with an emphasis on case study using observation, interview and reflective diary methods. Further areas for research include: the investigation of gendered approaches to creative collaboration, collaborative creativity, and collaborative creative learning; the use of more diverse research methodologies and methods and techniques including large-scale quantitative studies and arts-based and arts-led approaches; and the investigation of more diverse music settings.

## Background

Studies of creativity emerging from cultural psychology (Glaveanu, 2010a,b; Barrett et al., 2014; Glaveanu et al., 2014) and social psychology theoretical frameworks (Miell and Littleton, 2004; MacDonald et al., 2005) increasingly challenge individualist conceptions of creativity to argue that social interaction, communication, and collaboration are key elements in creative thought and practice. Vera John -Steiner's seminal work *Creative Collaboration* published in 2000 identifies a number of contributing factors for the turn from an individualist Western focus on the solitary creative genius to a social constructivist view of creativity. These factors include the waning of Piagetian views of learning and development in the second half of the 20th century as Vygotsky's writings from the 1920–1930s Soviet era became known in the English-speaking world through translation (Vygotsky, 1978, 1986), and the take up of cultural psychology as a theoretical framework for learning and development (see for example Bruner, 1996; Cole, 1996). Whilst others had explored creativity as a social rather than individual phenomenon in earlier work (Amabile, 1983), arguably, the notion of active co-contribution to creative production was explored in depth for the first time in John-Steiner's volume.

John-Steiner's focus was on intellectual and artistic collaboration as evidenced in long-standing creative partnerships. Drawing on Howard Becker's notion of “art worlds” (Becker, 1982), she identified the ways in which complementarity, mutuality, interdependence, and joint activity underpinned creative work. For John-Steiner, “*humans come into being and mature in relation to others*” (2000, p. 187, John-Steiner's italics). Collaboration thus “…provides a mutual zone of proximal development where participants can increase their repertory of cognitive and emotional expression” (p. 187). John-Steiner presents a model of creative collaboration which identifies four patterns of collaboration: distributed, complementary, family, and integrative. Distributed collaboration is that which occurs in shared thought communities, or loose networks of collaborative groups, where ideas and practices may be shared and appropriated for individual as well as for collective ends. Complementary collaborations rest in the recognition and instrumentalization of complementary expertise, disciplinary knowledge, roles and temperaments to pursue a common goal (2000). Family collaborations, whilst nested in the notion of familial relationships (e.g., life-partners), focus on the ways in which relationships, roles and responsibilities may shift between members over time and between tasks. Importantly, these collaborations rely on a heightened sense of mutual obligation, shared companionship, and belonging, as well as a capacity to survive or manage productively the tensions, conflicts, and disagreements that might arise through collaborative work. John-Steiner's fourth pattern of collaboration, integrative collaboration is created in and built upon joint endeavors to effect “transformative change.” She emphasizes that these four patterns of creative collaboration are not hierarchical; rather, they serve different ends in producing creative work.

Whilst early investigations of creative collaboration emphasized the role of social interaction in creative collaboration (John-Steiner, 2000), more recent work in creative collaboration has expanded the notion of “distributed creativity” (Glaveanu, 2014) to encompass interactions between creator and audiences, materials, embodied actions, and the historico-socio-cultural affordances of the creative activity and environment. This approach simultaneously expands the potentialities of creative collaboration beyond instances of direct human interaction and engagement and reminds us that multiple human interactions at various removes across time and space underpin any creative endeavor.

In the above we have focused on creative collaboration as the key concept. Whilst collaborative creativity might be viewed merely as a synonym for creative collaboration, the reversal of emphasis may offer opportunity for differing perspectives to emerge. For example, in foregrounding the term “collaborative” the emphasis is placed on the role of the groups and teams (Sawyer, 2017; Paulus and Nijstad, 2019) in producing creative outcomes rather than the outcomes themselves. Research in this area, often undertaken in industry and innovation contexts (Mumford, 2012), seeks to identify the intra- and inter-personal, environmental, and socio-cultural factors that contribute to effective teamwork, group and organizational creativity.

Delalande, whilst acknowledging the “eminently solitary” nature of “Western erudite music” reminds us that music creation also has a long history of collaborative practice. He notes

…throughout the time when the technology of writing dominated the practice of Western erudite music—roughly since the 13th century—creation was an eminently solitary activity, which has not been the case within the oral tradition (since creation in the oral tradition comes about in the very course of transmission). (2016, p. 457)

He writes of the “compelled cooperation” of teams, whether “direct or indirect” (what might also be viewed as distributed), illustrating the work of collaborative creative teams in music through reference to the work of GRM (Groupe de Recherches Musicales) and IRCAM (Institute de Recherche et Coordination Acoustique/Musique). This work is described as respectively “cooperation” between several composers, between tool developers and composers, and, between musical assistants and composers. Whilst the focus here is on composer-focused collaborative creativity, others recognize the roles of performers including performers and conductors (Ravet, 2016), performers and composers (Bayley and Lizée, 2016), and performers and audiences (Freeman and Godfrey, 2010) as sites for collaborative creativity. This body of research demonstrates an increasing interest in collaborative creative music practices in the western classical music profession, and a move away from the notion of creativity being the preserve of the solitary genius (Sawyer, 2017).

We suggest that music performance, improvisation and composition may be viewed as exemplary “laboratories” of creative collaboration and/or collaborative creativity through the historico-socio-cultural affordances (or constraints) they offer and the combined elements of audiences, materials, embodied actions and the collaborative teams that are involved (composers, performers, conductors, tool developers, music assistants etc.). It is notable that the research “laboratories” cited above have focused largely on “eminence” settings (see Gardner, 1993; Csikszentmihalyi, 1996), that is, those settings which provide a means to tap the knowledge and expertise of leaders and professionals in a specific field (Barrett, 2006; Barrett and Gromko, 2007). Ericsson endorses this approach and “…rejects the assumption that data on large samples of beginners can be extrapolated to samples of elite and expert performers” (Ericsson, 2014, p. 81). He argues for “expert-performance” approaches to investigations of advanced skills and knowledge. Accordingly, the study of these laboratories raises possibilities not only for the discipline of music but also holds potential for other domains of creative collaborative practice. In what follows we report the findings of a systematic literature review of collaborative creativity and creative collaboration in music, focusing on “eminence” settings of practice including teaching and learning. Our investigation was guided by the following questions:

(1) How and in what eminence music settings has creative collaboration or collaborative creativity been investigated?
(2) What problems and research questions have been the focus of research in those settings?
(3) How are creative collaboration and/or collaborative creativity described, defined and framed in eminence music settings?
(4) What are the practical implications of research concerned with creative collaboration and/or collaborative creativity in eminence music settings?

## Methodology

Our approach to conducting the systematic literature review was guided by the PRISMA (Preferred Reporting Items for Systematic Reviews and Meta-Analyses) checklist (Moher et al., 2009). This provides guidelines for developing search protocols, searching data bases, selection of studies, analysis of relevant characteristics, and synthesis of results. The full-text articles were coded using SPIDER [Sample, Phenomenon of Interest, Design, Evaluation (i.e., key findings), Research type] tool developed by Cooke et al. (2012) for synthesis of qualitative evidence. In this section of the paper we provide an outline of our use and implementation of this approach to a systematic literature review, including the development of the search protocol and the procedures for first and second screening.

### Developing the Search Protocol

Prior to undertaking the search of data bases, and in accordance with an iterative approach (Moher et al., 2009), an exploratory search was undertaken to ascertain the timeline for when the research on the topic has been published. Using a university library search engine keywords “creative collaboration,” “collaborative creativity,” “collaboration,” and “creativity” were combined with “in music” and with “and music” to explore *when* academic publications on this topic began to appear. In addition, the team had brainstormed possible keywords for the main search and these were confirmed by the exploratory screening. The following search protocol for the main search was adopted:

Line 1—(Collaborat^*^ or team^*^ or share^*^ or reciproc^*^ or mutual^*^ or intersubjectiv^*^ or collective or empath^*^ or entrain^*^ or attun^*^ or system^*^ or group or ensemble or social or distributed).
Line 2—(creative^*^ or new or innovat^*^ or original^*^ or novel^*^ or problem-solv^*^ or problem-find^*^ or flow or improv^*^ or emergent).
Line 3—(pedagog^*^ or apprentice^*^ or leader^*^ or mentor^*^ or guid^*^ or teach^*^ or learn^*^ or practice or master^*^).
Line 4—(music).

### First Search and Screening

Three data bases (Web of Science, ERIC and JSTOR) were searched using the combined keywords of the search protocol, in English, published between 1990 and 2021, searching under Topic/All fields for peer-reviewed journal articles with output by relevance. Parameters were set in order to limit the results to papers published in English as the shared language of all team members; published since 1990 because exploratory screening had identified no publications before 1990 and only a handful of papers in the 1990s, and with a cut-off at 2021 as the date when this research was carried out; searching under “Topic (Web of Science)/All fields (JSTOR)/Search anywhere (ERIC)” was adopted when an “Abstract” search resulted in zero outputs in some data bases; book chapters were eliminated from the search as these frequently synthesize existing literature rather than report on new research, with peer-reviewed articles typically undergoing a more stringent peer review process.

The search identified 6,347 items and after the first screening and removal of duplicates, 138 items were deemed relevant according to the first screening criteria.

First screening criteria:

- In English;
- Published between 1990 and 2021;
- Peer-reviewed journal articles;
- Context of music;
- Creativity and/or collaboration as either key concept.

### Second Screening

**Figure 1:**
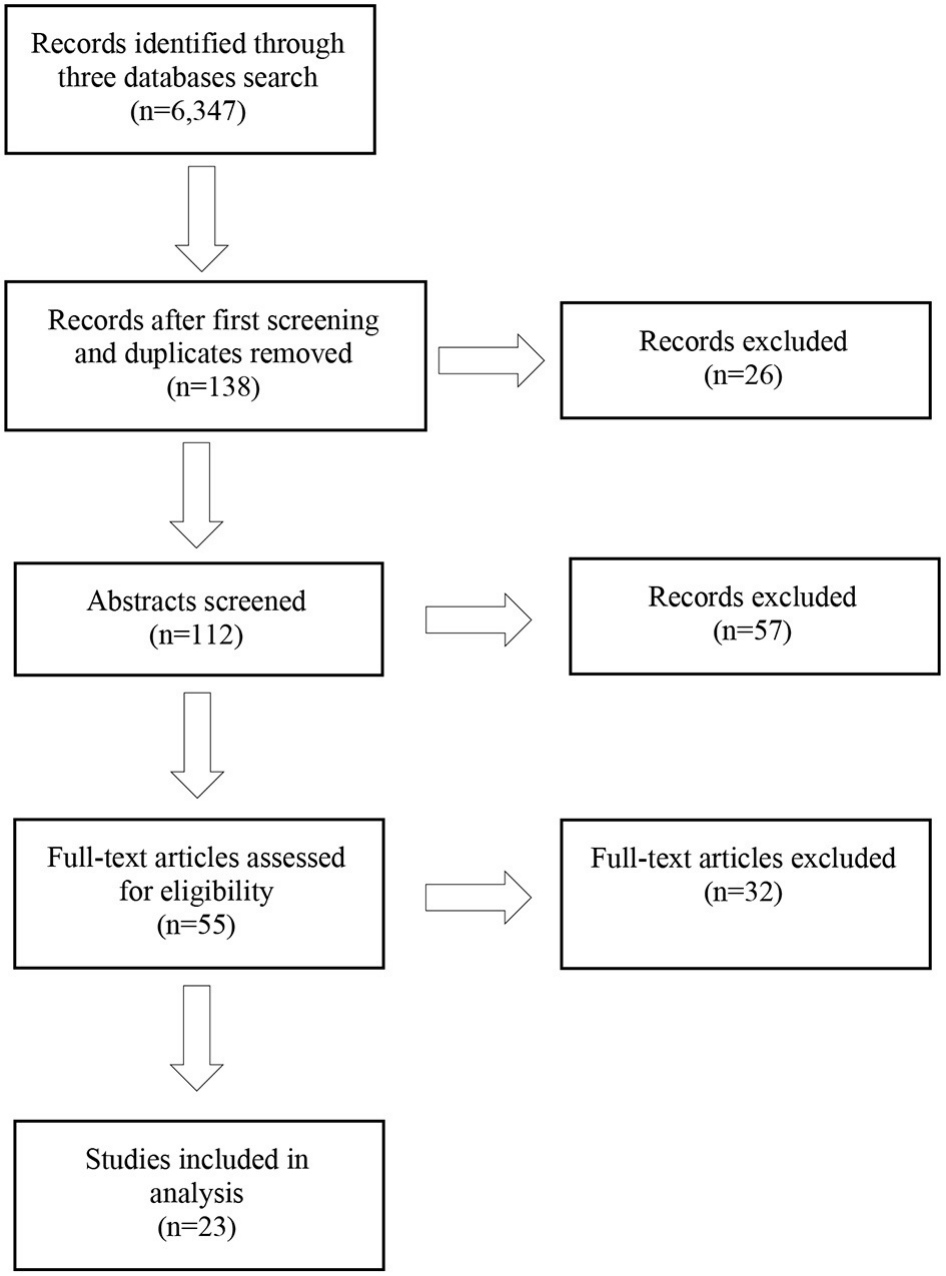
Flow-chart of the screening process.

Abstracts of the 138 retained articles were screened by one team member and main points summarised under the following topic areas: composition (technology and traditional methods), teacher education/higher education/professionals, theoretical papers, inter-disciplinary papers, jazz/popular music, brain studies, community music, and improvisation. Papers concerned with primary or secondary school contexts were excluded (n = 26) due to our focus on eminence settings, leaving 112 papers (see Table 1 and Figure 1).

**Table 1:** Screening criteria.

	**Search engine**	**Keywords**	**Language**	**Dates**	**Filters**
Exploratory Search	University library	• Collaborative creativity and music • Collaborative creativity in music • Creative collaboration and music • Creative collaboration in music • Creativity in music • Collaboration in music	English	1980–2021	• Peer-reviewed journals • Journal articles • Books • Book chapters • By relevance
First search and screening	• Web of Science • ERIC • JSTOR	Lines 1–4 of search protocol combined	English	1990–2021	• Topic/All fields/Search anywhere • Peer-reviewed articles • Music context • Creativity and collaboration as either key concept
Second screening			English	1990–2021	• Abstract screening • Exclude primary and secondary education

### Retained Papers

The full team (three researchers) screened the 112 retained papers (titles and abstracts only) independently and after discussion 57 papers were excluded according to the following criteria for retention:

- Context: Professional training and practice in music performance, improvisation and composition, where “creative collaboration” or “collaborative creativity” is a primary focus
- Peer-reviewed journal article
- In English
- Empirical study
- Both key concepts of creativity and collaboration are the focus of study

Papers were excluded at this stage if all three team members agreed. Papers where there was disagreement were retained at this stage.

An analysis of the full texts of the remaining 55 articles was carried out. The articles were read by the researchers and coded using the SPIDER tool developed by Cooke et al. (2012) (see Table 2). The original categories were adapted to the music context through iterative team discussions during the coding process.

**Table 2:** SPIDER tool [adapted from Cooke et al. (2012) to the study's context].

**SPIDER**	**Justification**
S—sample	Participants in higher education, professional development, professional training, and practice in music performance, improvisation, and composition, where “creative collaboration” or “collaborative creativity” is a primary focus
PI—phenomenon of interest	Creative collaboration or collaborative creativity in music
D—design	Methods: establishing credibility, transferability, reliability, and validity issues
E—evaluation	Outcomes/Key findings: music processes; interpersonal processes; intrapersonal processes; pedagogy and facilitation; definitional
R—research type	Qualitative or Quantitative or Mixed methods (open) paradigm

Theoretical papers (*n* = 5) were retained for the Background section and only empirical papers were considered for full-text analyses (*n* = 23) (see Figure 1).

Twenty-three papers were retained for analysis, while 32 were excluded. Five of these papers informed the theoretical background to the systematic review but were not included in the analysis as they were not empirical papers. Reasons for exclusion from analysis were: exclusive focus on one concept (either creativity or collaboration) rather than the combined concept (creative collaboration or collaborative creativity) and non-empirical papers.

Publication years ranged from 2006 to 2020. Seven studies were carried out in Australia and six studies were carried out in the UK. A further three studies were undertaken in the USA and one in Canada. Six studies were undertaken in European countries: Italy (two studies), Denmark, Finland, Ireland and Spain, and one in Singapore (Table 3).

**Table 3:** Geographical distribution of papers retained for analysis.

	**Region**							**Total**
	**Australia**	**Canada**	**USA**	**Europe**	**Asia**	**UK**	
Publication year	2006	1	0	0	0	0	1	2
	2007	0	0	2	0	0	0	2
	2011	0	0	1	0	0	0	1
	2012	2	0	0	0	0	1	3
	2014	0	0	0	2	0	0	2
	2015	0	0	0	1	0	1	2
	2016	1	0	0	1	0	1	3
	2017	0	0	0	1	0	1	2
	2018	1	1	0	1	0	0	3
	2019	2	0	0	0	0	0	2
	2020	0	0	0	0	1	0	1
Total		7	1	3	6	1	5	23

### Addressing Issues of Quality and Bias

As noted above the analysis was guided by the PRISMA checklist (Moher et al., 2009). We also drew on the JBI Systematic Reviews Checklist for Qualitative Research (Joanna Briggs Insitute, 2017) to address issues of quality and bias. In accordance with both these guidelines, we worked as a team of three, with each member reading every paper and subsequently discussing each paper (via regular zoom meetings), in order to establish that we were in agreement that each paper met our inclusion critieria. Given that 22 of the 23 papers retained for analysis were qualitative studies our critical appraisal of the papers focused on the qualitative markers of methodological rigor, including credibility, transparency and trustworthiness (Lincoln and Guba, 1985), and examined congruity between philosophical perspective and research methodology, methodology and research questions and objectives, methodology and methods, representation and analysis of data, and interpretation of results (Joanna Briggs Insitute, 2017). Discussions also addressed issues of researcher reflexivity, participant voice, ethical processes, and conclusions.

Some methodological limitations among the retained papers were noted (Table 4). For example, while all 23 of the papers provided sufficient detail about the cultural and theoretical location of the research, four of the 23 papers did not discuss explicitly the influence of the researcher in the interpretation of the data. A further five papers, while not explicit in discussing this point, did address this issue by demonstrating how findings had been triangulated. For three practice-based artistitic research studies this issue was deemed to be not applicable. One methodological concern among this group of papers rests in the ways in which ethical issues were reported. Only six papers included explicit detail concerning ethics review board approval or informed consent procedures. However, a further 17 papers did provide sufficient information to be able to ascertain that the study had been carried out in an ethically responsible manner, particularly with respect for consent, confidentiality and anonymity. One further potential limitation of the findings reported lies in the small sample sizes which are a feature of qualitative approaches. Nevertheless, such studies offer a depth and richness of data and analysis that yields findings that may be transferred to other settings.

**Table 4:** Criteria for assessment of methodological rigor.

	**Number of papers in each evaluation category**			
**Criteria**	**Yes**	**No**	**Not clear**	**Not applicable**
Congruity: philosophical perspective with methodology	23			
Congruity: methodology with research question	23			
Congruity: methodology with data collection methods	23			
Congruity: methodology with analysis and representation of data	23			
Congruity: methodology with interpretation of results	23			
Researcher positionality: cultural and theoretical location of research	23			
Influence of the researcher	11	4	5	3
Participant voice represented	22			1
Ethics	6		17	

In addition to the appraisal points reported in Table 4, the retained quantitative papers were deemed to include sufficient information when evaluated against a further set of criteria that included: (1) criteria for inclusion of participants; (2) the context explained fully; (3) the reliability and validity of approaches to measurement; (4) transparency with regard to confounding variables; (5) appropriate statistical analyses. These papers were found to fulfill each one of these criteria.

Notwithstanding the noted limitations, the researchers reached consensus in each instance, agreeing that each paper was sufficiently rigorous for inclusion in the review. Finally, as a further consideration of quality, through establishing publication in a refereed journal as an inclusion criterion, each article retained for analysis had already been submitted to a rigorous quality appraisal through the academic peer review process. Therefore, no further papers were excluded following critical appraisal of the methodological rigor.

## Findings

### How and in What Eminence Music Settings Has Creative Collaboration or Collaborative Creativity Been Investigated?

#### Settings

Eight studies took place in higher education music disciplines including performance (one study), improvisation (four studies), composition (two studies) and recording studio practice (one study). Fourteen studies were carried out in professional music contexts, with eight of those concerned with an analysis of professional practice and five focused on processes associated with professional development. Among the professional development studies, two focused on developing expertise in improvisation, two focused on composition and one was undertaken in the context of recording studio practice. Finally, two further studies took place in community settings, where the focus was on improvisation (Table 5).

**Table 5:** Settings for the research.

		**Performance**	**Improvisation**	**Composition**	**Recording studio practice**	**Total**
Setting	Higher education	1	4	2	1	8
	Professional development	0	2	2	1	5
	Professional practice	3	4	1	0	8
	Community	1	1	0	0	2
Total		5	11	5	2	23

Table 6 shows the musical genres that characterized the research studies. The greatest number of studies were carried out in the context of jazz (10 studies), with seven out of those 10 studies located in professional contexts. In contrast, world music was represented in just one study carried out in a community context. Five studies concerned Western classical music-making, with two of those focusing on professional performance and a further three focusing on improvisation or composition in higher education settings. Two studies were carried out in the context of popular music in higher education, while a further four studies focused on popular music in professional contexts. Finally, contemporary art music formed the context for two further studies, one focusing on improvisation and the other on performance.

**Table 6:** Musical genres represented in the research.

**Setting**		**Musical genre**					**Total**
		**Contemporary**	**Popular**	**Jazz**	**Western classical**	**World**	
HE	Performance	0	1	1	0		2
	Improvisation	1	0	2	1		4
	Composition	0	0	0	2		2
	Recording studio practice	0	1	0	0		1
	Sub-Total	1	2	3	3		9
Professional development	Improvisation		0	2			2
	Composition		2	0			2
	Recording studio practice		1	1			2
	Sub-Total		3	3			6
Professional practice	Performance	1	0	0	2		3
	Improvisation	0	0	4	0		4
	Composition	0	1	0	0		1
	Sub-Total	1	1	4	2		8
Community	Performance			0		1	1
	Improvisation			1		0	1
	Sub-Total			1		1	2
	Total	2	6	11	5	1	25^*^

* *Total is >23, because some studies were multi-genre*.

#### Sample

Twenty-two of the 23 studies reported their sample size, and overall, this ranged from 1 to 64, with a mean sample size of 13.86. Just seven studies reported the ages of their participants, which overall ranged from 17 to 55. The mean participant age range among those seven studies was 27–39. Fourteen studies reported that their research included male participants, while 10 of those 14 studies also included female participants. Overall, of the 14 studies that reported the sex of their participants, a total of 72 males were included in the research compared with 32 females.

### What Problems and Research Questions Have Been the Focus of Research in Those Settings?

#### Professional Development

Among the studies concerned with professional development, Biasutti (2015, 2018) used video observations and in-depth interviews to explore the development of expertise in collaborative online composition. This study analyzed communication modes and learning strategies among three professional electronic band musicians who had previously only collaborated in offline environments. Likewise framed with a strong focus on professional development, Brinck (2017) used ethnographic methods to capture how learning to jam can emerge through situated learning practice. In a similar vein, de Bruin (2016, 2019) used a phenomenological approach to explore elite improvisation performers' lived experiences of evolving creative improvisation practices.

#### Professional Practice

Studies of professional practice differed from those exploring professional development through their emphasis on understanding the nature of creative collaborative processes rather than on the practices that supported learning. For example, Hill and Fitzgerald (2012) adopted a participant-observation approach in exploring creative professional practice, with a focus on understanding how musical and interpersonal interactions among live electronica musicians contributed to a coherent musical performance. Also, in the context of performance, the interpersonal dimensions of control and trust were the focus of Khodyakov's (2007) study that used in-depth interviews and observations to explore the creative and collaborative professional practices within a conductorless contemporary orchestra. With a similar focus on the intersection of interpersonal interaction with collaboration and creativity in professional practice, Hill et al. (2018) carried out a reflective, participant observation analysis of the role of conflict among band members engaged in collaborative composition.

Morgan et al. (2015) investigated interpersonal behaviors in the context of professional improvisation. In this study, the researchers used electronic sensors, video and self-report measures to explore relationships between non-verbal behaviors (e.g., gaze, posture), physiological response (e.g., heart rate), and facets of the musical process such as creative decisions. Musical interactions within ensembles, as compared with solo settings, were explored by Marchini et al. (2014), who used computer modeling to investigate how inter-voice dependence may be related to musical expressivity. Taking a slightly different approach to exploring professional practice, other researchers (MacDonald and Wilson, 2006; Wilson and MacDonald, 2012, 2017) have interrogated the way professional jazz musicians use language (in the context of interviews and focus groups) to construct musician identity and a professional discourse about improvisation.

#### Higher Education

Among the studies in higher education, seven were carried out with undergraduate students, while just one was carried out in a Doctoral Studies program, focusing on recording studio practice.

Within higher education, van Nort (2018) carried out a piece of practice-based research using participant observation to explore intersubjectivity within an electroacoustic orchestra performance where the music-making was guided by a form of improvised conducting known as Sound-painting. With a similar interest in electronic music contexts, Freeman and Van Troyer (2011) explored processes associated with real-time creativity, or the fusion between improvisation and composition. Using a process known as Laptop Orchestra Live Coding, this practice-based study analyzed musical interactions represented in text-based computer code among members of a laptop orchestra.

Collaborative learning within intensive workshops has been explored. For example, situated and collaborative learning experienced within jazz and popular music conservatoire performance workshops was investigated by Virkkula (2016). Using a case study approach and gathering data via students' reflective journal entries, Virkkula investigated the processes of sociocultural learning within workshops structured as communities of musical practice comprising students and professional musicians. In a similar vein, de Bruin et al. (2019) explored collaborative learning that emerged from authentic, “real-world” rehearsal, workshop and performance opportunities where jazz students collaborated alongside professionals. The researchers used in-depth interviews pre- and post-three rehearsal/workshop/performance cycles, prompting students to reflect on the role of collaboration in their learning. Collaborative learning was also investigated by Blom (2012), who, in this instance, used open-ended questionnaires to gather insights about the learning that occurred within interdisciplinary (music, dance, drama) and collaborative improvisation workshops.

One-to-one contexts have also been the focus of research concerned with collaborative creativity and collaborative creative learning, in higher education. For example, learning and teaching in creative composition, occurring within one-to-one dyads have been explored (Barrett, 2006; Barrett and Gromko, 2007) using in-depth interviews and video observation to explore the collaborative processes between student-composer and composer-teacher. One-to-one peer learning in composition was the context for a subsequent study carried out by Dobson and Littleton (2016). In this study, video and audio recordings of the students' collaborative work was analyzed, looking at micro-moments where “collaborative conceptual creative themes” (p. 337) were articulated.

#### Community Contexts

Finally, two studies were carried out in community contexts. In the first (Kenny, 2014), the research adopted a participant observer role over a 9-month period, and gathered observations, interviews and reflections concerned with collaborative creativity in the context of a non-formal jazz ensemble, where adult participants were supported by eminent expert tutors. The second community-based study (Tan et al., 2020) used in-depth interviews to explore the relevance of creative collaboration in relation to the phenomenon of flow, as experienced by adult participants of a non-formal gamelan ensemble.

#### Research Designs

Eighteen of the retained studies were designed within a qualitative paradigm, including ethnography (1), case study (11), qualitative exploratory (2), practice-based artistic research (2), and phenomenological studies (2). Two studies were classified as quantitative, and three used mixed methods (Table 7).

**Table 7:** Research designs and paradigms.

	**Correlational**	**Ethnography**	**Case study**	**Qualitative exploratory**	**Practice-based artistic research**	**Phenomenology**	**Total**
Qualitative	0	1	11	2	2	2	18
Quantitative	1	0	1	0	0	0	2
Mixed	0	0	2	0	1	0	3
Total	1	1	14	2	3	2	23

#### Research Methods

Among the 23 retained studies, the most frequently used method was semi-structured interview (15 studies), followed by observations (11 studies). In addition, data were gathered through participant observation (five studies), journal reflections (four studies), and audio recordings (four studies). Finally, questionnaires and focus groups were each used in two studies, while computer code analysis was used in one study. Table 8 shows the methods used within each setting and area of musical practice. In higher education, in-depth semi-structured interviews (four studies), observation and journal reflections (three studies each) were used to the greatest extent. Within professional settings observations and participant observations were used in a total of 11 studies, compared to a total of nine studies using semi-structured interviews.

**Table 8:** Research methods used within settings.

		**Performance**	**Improvisation**	**Composition**	**Recording studio practice**	**Total number of studies using each method**
Higher education	Observation (including video)			2	1	3
	Semi-structured interview		1	2	1	4
	Journal and reflection	1	2			3
	Questionnaire		1			1
	Focus Group		1			1
	Participant observation		1			1
	Computer code analysis		1			1
	Sub-Total	1	7	4	2	14
Professional development	Observation (including video)			2		2
	Audio recording				1	1
	Semi-structured interview		2	2	1	5
	Participant observation				1	1
	Sub-Total		2	4	3	9
Professional practice	Observation (including video)	1	3	1		5
	Audio recording	1	1	1		3
	Semi-structured interview	2	2			4
	Questionnaire		1			1
	Participant observation	2		1		3
	Sub-Total	6	7	3		16
Community	Observation (including video)		1			1
	Semi-structured interview	1	1			2
	Focus group		1			1
	Journal reflections		1			1
	Sub-Total	1	4			5
	Total number of methods used in the 23 studies					43^*^

* *The total number of methods used is >23 because 16 studies used more than one method*.

Across all of the settings, the methods used were primarily qualitative, with the exception of one self-report rating scale questionnaire (Morgan et al., 2015), statistical analyses of features of expressivity extracted from multimodal recordings (Marchini et al., 2014) and quantitative analysis of computer code (Freeman and Van Troyer, 2011). Among the qualitative studies, approaches to analysis included thematic analysis (e.g., Barrett, 2006; Barrett and Gromko, 2007), discourse analysis (MacDonald and Wilson, 2006; Wilson and MacDonald, 2012; Dobson and Littleton, 2016), content analysis (Freeman and Van Troyer, 2011; Virkkula, 2016; de Bruin et al., 2019), interpretative phenomenological analysis (IPA: Wilson and MacDonald, 2017) and finally, the constant comparative method (Blom, 2012; Biasutti, 2015, 2018; Hill et al., 2018).

### How Are Creative Collaboration and/or Collaborative Creativity Described, Defined, and Framed in Music Settings?

#### Phenomenon of Interest

The phenomenon of interest in each paper was examined and coded according to the relative focus on the core concepts of collaboration and creativity. Six papers were framed with a focus on creative collaboration, while a further 10 papers were framed by the idea of collaborative creativity. Eight papers were primarily concerned with collaborative creative learning. Of those concerned with *collaborative creative learning*, six studies were carried out in higher education (Barrett, 2006; Barrett and Gromko, 2007; Blom, 2012; Dobson and Littleton, 2016; Virkkula, 2016; de Bruin et al., 2019) and two in the context of professional development (de Bruin, 2016; Brinck, 2017). Where the phenomenon of interest was conceptualized as *creative collaboration*, one study was carried out in the context of higher education (van Nort, 2018), four studies were located in professional practice contexts (Khodyakov, 2007; Hill and Fitzgerald, 2012; Morgan et al., 2015; Hill et al., 2018), and one was undertaken in a community context (Tan et al., 2020). Finally, among the papers where *collaborative creativity* was the core concept, one study was located in higher education (Freeman and Van Troyer, 2011), three were concerned with professional development (Biasutti, 2015, 2018; de Bruin, 2019), five were concerned with professional practice (MacDonald and Wilson, 2006; Wilson and MacDonald, 2012, 2017; Marchini et al., 2014) and one study was located in a community context (Kenny, 2014) (Table 9).

**Table 9:** Phenomenon of interest in each setting.

	**Higher education**	**Professional development**	**Professional practice**	**Community**	**Total**
Creative collaboration	1	0	4	1	6
Collaborative creativity	1	3	4	1	9
Collaborative creative learning	6	2	0	0	8
Total	8	5	8	2	23

#### Theoretical Frameworks

Papers were coded in the *creative collaboration* category when their focus was on collaborative processes, with creativity embedded within the collaboration. Studies within this category drew upon sociocultural perspectives (e.g., Khodyakov, 2007), the theory of flow (e.g., Morgan et al., 2015; Hill et al., 2018; Tan et al., 2020) and intersubjectivity (e.g., van Nort, 2018). Key facets of creative collaboration, as discussed in this group of papers, were non-hierarchical approaches, a collectivist mindset where all members of the group have “equal contributional potential” (van Nort, 2018, p. 68) and unpredictable outcomes, alongside the idea of emergent intentionality in creative practice unfolding over time. A feature of creative collaboration is that “the nature and quality of the interactions between ensemble members is a critical determinant of musical outcomes” (Hill and Fitzgerald, 2012, p. 169), or as discussed by Tan et al. (2020), the intersection of relationship, community and peak musical experiences. These intersections may be framed with what Khodyakov (2007, p. 7) refers to as the “chamber paradigm,” guided by “the principles of collaboration, equality and democracy” and occurring within a musical context where creative decision-making is distributed among the group. Hill et al. (2018, p. 195) draw attention to “empathetic attunement,” as proposed by Seddon (2005), occurring when musicians are able to adopt the perspectives of their co-performers. Similarly, “parallel processing (simultaneous awareness of self and collaborators),” emotional contagion and behavioral mimicry have been highlighted as characteristics of group flow in creative collaboration (Morgan et al., 2015, p. 33). In this vein, creative collaboration may be akin to “improvisational creativity as it manifests in collective musical performance” (van Nort, 2018, p. 68).

Papers coded in the *collaborative creativity* category were primarily focused on creative processes or outcomes, with collaboration positioned as an intersecting process. Within this category, studies drew on sociocultural perspectives (e.g., Biasutti, 2015, 2018), social constructivist perspectives (e.g., Kenny, 2014), and discursive psychology (e.g., Wilson and MacDonald, 2012), theory of flow (e.g., Marchini et al., 2014) and coregulation (e.g., de Bruin, 2019). Here, the social dimension was conceptualized as being central within the creative process (MacDonald and Wilson, 2006), embedded within “multiple practices and multiple creativities corresponding to music's social and technological mediations” (de Bruin, 2019, p. 30). For example, Freeman and Freeman and Van Troyer (2011, p. 11) describe creative processes as “conversational interactions.” Wilson and MacDonald (2012) refer to a spontaneous process characterized by non-verbal interaction, later (Wilson and MacDonald, 2017, p. 137) emphasizing “social creativity” underpinned by shared understandings and mutual engagement. Similarly, Kenny (2014) conceptualizes collaborative creativity as contextualized and communicative, founded upon social and collective processes. As described by Biasutti (2015, p. 118), “the social dimension is intrinsic to creativity and creativity is embedded in interaction.”

Within the creative, expressive elements of the music itself, collaborative creativity may be conceptualized as the interplay between “polyphonic expression (each musician plays their melody with possibly a different expression in respect to the one of the other concurrent voices) and inter-dependence among musicians (each musician takes into account information about concurrent voices to shape their expression)” (Marchini et al., 2014, p. 304). Furthermore, in addition to “musical and social practices,” sustained engagement in collaborative musical creative practices may involve “leadership and participatory membership and a challenge” (Biasutti, 2018, p. 475).

*Collaborative creative learning*, as conceptualized in the papers reviewed, could be traced to sociocultural perspectives on learning (e.g., Barrett, 2006; de Bruin, 2019) where, for example, the development of our “highest mental functions” (Barrett, 2006, p. 198) and the related phenomenon of qualitative transformations in understanding emerge from systematic and sustained cooperation between students and teacher. In this way, the development of competence may be seen as a construction of new skills and knowledge through a communal process in “communities of practice” (Virkkula, 2016, p. 28; Brinck, 2017).

Within this collaborative creative learning category, studies drew upon the idea of eminence (e.g., Barrett, 2006), exploring “the ways in which the creative artist engages with the social and cultural institutions of his or her environment through the use of cultural tools and social practices developed in that environment” (Barrett, 2006, p. 198). The studies in this category were furthermore guided by theoretical ideas relating to distributed collaboration (e.g., Blom, 2012), communities of practice (e.g., Brinck, 2017) and flow (e.g., Virkkula, 2016). Accordingly, collaborative learning was conceptualized as an “emergent group property” (Blom, 2012, p. 725) that is dependent upon the nature of social relationships as pathways “toward deep engagement in learning” (de Bruin et al., 2019, p. 1). In a similar vein, Dobson and Littleton (2016, p. 334) highlight the related idea of “collaborative emergence” where actions and interactional consequences exist in a contingent relationship that may lead to unpredictable learning outcomes, and where learning processes are collaborative in the sense that each participant contributes equally.

### What Are the Practical Implications of Research in Creative Collaboration or Collaborative Creativity Within Eminence Music Settings?

Overall, the 23 retained papers contributed key findings concerned with facets of musical, interpersonal and intrapersonal processes found to be associated with creative collaboration, collaborative creativity, and pedagogies of collaborative creative learning (Table 10). Facets of musical processes that emerged included the ideas of fusion (e.g., improvisation and composition), “pace” (i.e., a slow and evolving process occurring over time, or alternatively a rapidly paced and immediate phenomenon occurring in the moment of performance), and code systems or signifiers. Many papers highlighted the interplay between social and musical processes, positioning collaboration as a central characteristic of creative practice (e.g., Biasutti, 2015, 2018), or situating creativity as being embedded within collaboration (e.g., Kenny, 2014). Furthermore, findings from some studies pointed to the relevance of intrapersonal processes such as identity work, and the ways in which that intersected with musical and social facets of creative collaboration. Finally, a group of papers contributed to our knowledge relating to pedagogical principles and practices that frame collaborative creative learning, while similarly illustrating the key role that interpersonal issues play in mediating the relationship between collaboration and creativity.

**Table 10:** Key findings.

		**Music process**	**Intrapersonal**	**Interpersonal**	**Pedagogy**	**Total**
Creative collaboration	Hill and Fitzgerald (2012)	1		1		2
	Hill et al. (2018)	1		1		2
	Khodyakov (2007)	1		1		2
	Morgan et al. (2015)	1		1		2
	Tan et al. (2020)	1		1		2
	van Nort (2018)	1		1		2
	Sub-Total	6		6		12
Collaborative creativity	Biasutti (2015)	1		1		2
	Biasutti (2018)	1		1		2
	de Bruin (2019)	1		1		2
	Freeman and Van Troyer (2011)	1		1		2
	Kenny (2014)	1		1		2
	MacDonald and Wilson (2006)	1	1	1		3
	Marchini et al. (2014)	1		1		2
	Wilson and MacDonald (2012)	1	1	1		3
	Wilson and MacDonald (2017)			1		1
	Sub-Total	8	2	9		19
Collaborative creative learning	Barrett (2006)			1	1	2
	Barrett and Gromko (2007)			1	1	2
	Blom (2012)			1	1	1
	Brinck (2017)	1		1	1	3
	de Bruin (2016)			1	1	1
	de Bruin et al. (2019)			1	1	2
	Dobson and Littleton (2016)			1	1	2
	Virkkula (2016)		1	1	1	3
	Sub-Total	1	1	8	8	18
	Total key findings	15	3	23	8	49^*^

* *This number adds to more than 23 because several papers contributed key findings in more than one category*.

#### Musical Processes

Key findings concerned with musical processes were reported in research focused on performance (e.g., Freeman and Van Troyer, 2011), composition (e.g., Hill and Fitzgerald, 2012; Hill et al., 2018), and improvisation (e.g., Blom, 2012; Virkkula, 2016; van Nort, 2018). For example, a fusion of composition and improvisation was found in the context of a laptop ensemble, where the substance of the musical improvisations was derived from a live coding process in which text messages were translated to rhythmic files and shared or further transformed over a local network (Freeman and Van Troyer, 2011). Exploring the collaborative creativity framing this process, Freeman and Van Troyer (2011) reported that the mediated improvisatory approach, involving live coding of text-based messages, fostered a slow pace and evolving process characterized by extensive use of looping and somewhat constrained risk taking.

Pace was found to be more direct and immediate when creative collaboration in an electronic music ensemble was framed by an improvisational form of conducting known as Sound-painting. Here a lexicon of gestures functioned as codes that indicated who should play what, as well as how and when it should be played. Writing about the musical process shaped by Sound-painting and mediated by machine performers as well as human performers, van Nort (2018, p. 72) explains that “in the moment” creative choices are guided by a coded system whereby “there exist a number of gestures in which continuous conductor action is directly reinforced, interpreted or reacted to by members of the ensemble.” A similar quick pace of creative collaboration was reported in the context of live electronic dance music (Hill and Fitzgerald, 2012), where the musical process was characterized by “an advanced ability to listen closely and react quickly and creatively in real time in order to create a coherent groove and satisfying musical whole” (p. 170). In this instance—and contrasting with the examples where gestural or text-based codes mediated the musical collaboration—the layered rhythmic structures and sound textures formed the code system that guided and shaped the evolving creative performance.

#### Interplay Between Social and Musical Processes

The closely enmeshed strands of musical and interpersonal processes have been highlighted in research concerned with creative collaboration (Khodyakov, 2007; Hill and Fitzgerald, 2012; Morgan et al., 2015; Hill et al., 2018; van Nort, 2018; Tan et al., 2020) and collaborative creativity (Freeman and Van Troyer, 2011; Kenny, 2014; Marchini et al., 2014; Biasutti, 2015, 2018; de Bruin, 2019). This intersection between musical and social facets of creative collaboration was evidenced by Morgan et al. (2015), who reported a link between timing synchrony and interpersonal eye contact among improvising drummers, as well as correlations between visual contact and self-reports of creativity and engagement. The interplay between musical and social processes was also reported by Kenny (2014) who highlighted “privileging improvisation, maintaining challenge, and building knowledge through leadership and collaboration” as key mechanisms whereby creative practice (in this case in the context of jazz) may be situated in collaboration. Social bonding and unity of purpose (a function of interpersonal processes) have been reported to be integral to the musical process (Hill and Fitzgerald, 2012; Tan et al., 2020). Unity or mutual understandings relating to the interwoven strands of social and musical processes were further illustrated by Khodyakov (2007) who reported that “successful performance [in a conductorless, democratic orchestra] requires both trust and control” (p.18). Creative collaboration was achieved outside of the limitations of hierarchical structures typically found in orchestras, instead being premised upon shared creative decision-making framed by mutual obligation and expectations, civility and leadership rotation.

Subsequently, Hill et al. (2018), analyzing examples of their own collective composition work that occurred over a longitudinal (2-year) project, reported conflict to be an integral step of a process that also included instruction, cooperation and collaboration. Moments of conflict were found to be followed by sustained periods of engagement in the task, where group flow and empathetic creativity emerged. A critical issue highlighted by this study was that conflict could function as a catalyst for a creative musical process experienced within the rehearsal space, but that this occurred within a well-established, “meta-narrative” of a collaborative musical relationship evolving over time.

Biasutti (2015, 2018) investigated professional musicians engaged in collaborative online composition, noting two overarching and intersecting categories of musical and social processes. Collaboration in both of those process domains was achieved through verbal as well as non-verbal communication, and was underpinned by individual accountability, a commitment to high quality work and cooperation at all stages of experimenting, listening/evaluating, constructing, playing, and dealing with technical issues. Finally, Marchini et al. (2014) used computer modeling to explore the expressive parameters of performance, comparing solo to ensemble (string quartet) conditions. Distinctive differences were found between the two conditions, suggesting that interpersonal processes influenced the expressive nature of the performance. However, there was also some evidence that the expressivity in the solo condition was to some extent shaped by the experience of having collaborated in the ensemble condition (participants were members of a group that performed together on a regular basis); in other words, the musical implications of interpersonal processes reached beyond in the moment transactions. Collectively, these papers raise critical questions about the relationship between the individual and the collective, between tradition and unpredictability, and between the musical and social processes that characterize collaborative creativity in music.

#### Interpersonal and Intrapersonal Issues

Interpersonal and intrapersonal intersections have been explored in relation to the emergent and situated creative practice of jazz improvisation (MacDonald and Wilson, 2006; Wilson and MacDonald, 2012, 2017; de Bruin et al., 2019). The deeply social nature of jazz improvisation is discussed extensively in this literature, which also highlights jazz improvisation as a context where musical identity work can be shaped (e.g., Wilson and MacDonald, 2012, 2017). For example, identity work was shaped by discourses of mastery (corresponding to an incremental theory of self, whereby individuals believe in their own capacity to develop) vs. mystery (corresponding to an entity theory of self, whereby individuals are likely to believe that musical talent is a fixed trait). Furthermore, identity as a member of the jazz community was found to be reinforced by discourses that positioned improvisation as a “conversation” or alternatively as a “transcendental” creative practice founded upon flow-like experiences “of submersion of self within [the] group” (MacDonald and Wilson, 2006, p. 73). Learning to be a Jazz musician was also discussed within a long-term framework of identity development and learning (de Bruin, 2016) characterized by overlapping phases of self-regulation, co-regulation, and socially shared regulation (de Bruin et al., 2019). An overarching message in the literature is that collaborative creativity may involve balancing on the one hand exploration, diversity and unpredictability with, on the other, trust, familiarity, and convention. A further overarching message is that musical interactions have been found to be inseparable from interpersonal issues. For example, musical signifiers such as the choice to be silent or to play could be interpreted in multiple ways, requiring co-improvisers to draw on shared knowledge and experience to interpret the intention behind these signifiers (Wilson and MacDonald, 2012). At the same time, musical expectations could be confounded or disrupted by unpredictable or unexpected musical exchanges; in these instances, tensions between certainty and uncertainty required flexible responses and a tolerance–or even celebration of—ambiguity. This flexibility in turn was found to be premised upon trusting relationships and familiarity established over time (Wilson and MacDonald, 2017).

#### Pedagogies of Collaborative Creative Learning

Several studies have interrogated the pedagogies that characterize collaborative creative learning. Findings from these studies highlight the themes of exploration, embracing diversity, learning in community and transformation of knowledge. This body of research raises critical questions about the nature of the collective practice itself, within which collaborative creative learning can occur (Brinck, 2017). In this vein, several authors discuss situated learning in community, where students make music alongside professionals (e.g., Virkkula, 2016; Brinck, 2017; de Bruin et al., 2019). Overall, these papers point to a view of collaborative creative learning as being deeply embedded in collective, improvisational practices that embrace diversity and unpredictability (Brinck, 2017). Specific processes by which collaborative creative learning could be nurtured were concerned with the “communication of masterful standards” (Brinck, 2017, p. 221), learning how to learn, socialization (e.g., positive expectations, shaping values, and orientations to creative practice) and role acquisition (Virkkula, 2016; de Bruin et al., 2019). Pedagogical approaches took the form of scaffolded interactions such as modeling; problem-finding and guidance toward collaborative solutions. A sense of mutuality and shared regulation, expressed as joint goals, shared resources and interdependent rewards, was achieved through perspective-taking; role swapping and boundary crossing; and the use of dialogue (verbal or musical) for co-construction of knowledge and navigating resistance to change. Such practices offered “numerous possibilities for (changing) participation” for students and professionals alike (Brinck, 2017, p. 221) and—by extension—could be responsive to the diversity and unpredictability that characterized the collaborative and creative work.

Similar pedagogical and interpersonal issues were identified in the more formal contexts of collaborative creative learning among eminent composer-teachers and undergraduate student-composers (Barrett, 2006; Barrett and Gromko, 2007). Here, the communities of learning were positioned as “thought communities” (Barrett and Gromko, 2007, p. 214) distinguished by joint effort and social support, yet also framed by disciplinary historical, cultural and social practices. Pedagogical approaches were non-linear and reciprocal and could be conceptualized on a continuum from cooperative to autonomous. For example, at the cooperative end of this continuum were instances of scaffolding whereby the teachers provoked students to describe and explain or used probing and questioning to guide students toward solutions. In contrast, autonomous pedagogical approaches occurred when teachers became “fellow travelers,” seeking unpredictable solutions, extending the boundaries of tradition, and creating an environment in which could be found “license to change” (Barrett, 2006, p. 202).

Finally, key findings relating to pedagogies of collaborative creative *peer* learning have been reported (e.g., Blom, 2012; Dobson and Littleton, 2016). Where Blom (2012) focused on interdisciplinary peer learning temporally and geographically located within a specific workshop context, Dobson and Littleton (2016) explored disciplinary-specific (digital composition) peer learning processes that occurred over time and within multiple private and social spaces. Notwithstanding these contextual differences, both papers illustrate the phenomenon of “disruption” that was noted in the papers concerned with jazz improvisation (e.g., Wilson and MacDonald, 2017) and the role that can play in creative learning. For example, Dobson and Littleton (2016) highlight that collaboration has the capacity to disrupt or confound familiar digital practices, potentially meeting resistance to change but also prompting “possibility thinking” whereby students consider questions of “what if …” and develop elaborate understandings of steps to take and potential outcomes. In a similar vein, Blom (2012) noted resistance to change when music students encountered collaborative and improvisatory practices that disrupted their familiar and more individualistic artistic approaches. Noting that music students were initially reticent in collaboration with peers from other arts disciplines, Blom also highlighted the possibilities of knowledge that emerged from the musicians' proximity to—and interactions with—their drama and dance peers for whom tensions between individuality and collaboration were comparatively less prominent. Both papers also indicate that peer learning in creative work involves using dialogue or artistic practice to develop a common knowledge about each other's preferences, experiences and anticipated outcomes. This dynamic and continually evolving knowledge base becomes the basis for generating, evaluating and negotiating ideas within a process that may fluctuate between being “homogeneous”—where each voice is equal—and “heterogeneous,” where a leader is acknowledged (Blom, 2012, p. 734).

## Discussion and Concluding Remarks

The findings presented above illustrate that eminence investigations of creative collaboration and collaborative creativity have been undertaken within a range of settings in higher education, professional development and professional music-making with a more limited focus on these concepts within research carried out in community music settings. Studies have been carried out in contexts representing a small range of musical genres, with the majority focussing on creative collaboration in jazz or popular music. Studies carried out within Western classical music contexts have focused primarily on improvisation or composition. Very little research has been undertaken outside of jazz, composition, contemporary electronic or digital music genres, or indeed Western musical contexts. Further research is needed to interrogate the relevance of conceptions of creative collaboration and collaborative creativity as presented in these papers across diverse cultural contexts and across multiple musical genres.

The majority of studies have been designed within a qualitative exploratory paradigm, primarily case study, and seek to interrogate interpersonal processes and behaviors, musical interactions and the use of language to construct shared understandings around the nature of collaboration and creativity in improvisation, composition and contemporary practices in electronic music. A range of methods have been used, with the most prominent methods being semi-structured interviews, observations and participant observation. Analyses of qualitative data were framed in a range of different ways, including thematic analysis, discourse analysis, content analysis, IPA and the constant comparative method. Whilst many studies employed more than one method, only three studies used quantitative approaches. We suggest that there is opportunity to develop more diverse research methods that move beyond the identification of individual elements of creative collaboration and collaborative creativity in order to understand the potential causes and effects of these phenomena. Further methodological diversity might also be explored through the use of practice-based and/or practice-led artistic research.

The phenomena of interest ranged across expected categories of creative collaboration and collaborative creativity, as these were the focus of the review. An emergent category was that of collaborative creative learning, reflected in the higher education (8) and professional development (5) settings in which the bulk of the studies were located. Those studies investigating collaborative creative learning focused largely on strategies for scaffolding new knowledge in situated learning settings casting the teacher variously as collaborator, guide, coach, mentor. Further research is needed to understand the relationships between the positioning of these roles and the levels of experience, skills and expertise manifest in the teaching-learning interaction. Further investigation is also warranted in understanding the ways in which peer-to-peer learning is facilitated in creative collaboration and collaborative creative music learning settings.

Emergent inter and intra-personal issues highlighted the elements of disruption, conflict, and pace as components of creative collaboration and collaborative creativity, suggesting that these are perhaps necessary intersecting points in the development of collaborative work. The identification of these elements returns us to John-Steiner's four patterns of creative collaboration: distributed, complementary, family, and, integrative. None of the studies included in this systematic literature review could be classified as a family collaboration in terms of a familial connection as described by John-Steiner. A small number of those studies undertaken in professional settings (e.g., Khodyakov, 2007; Hill and Fitzgerald, 2012) might be classified as complementary in that musical goals were realized through drawing on complementary expertise, discipline knowledge, roles and temperaments. The studies were largely tacet in acknowledging distributed creativity in both John-Steiner's sense of drawing on loose networks of collaborative groups, or Glaveanu's notion of interactions between creator, audiences, materials, embodied actions, and the historico-socio-cultural affordances of the creative activities, although these might be inferred. Implicit in a number of studies is the underlying importance of relationships across time, of familiarity, of shared experience, of habitual patterns of work, and shared knowledge and experience that functions in a tacet way as a unifier (socially and aesthetically). It is also salient to note that John-Steiner's work emerged from a feminist paradigm, exploring theories of relational dynamics and gendered issues of ownership. Of the 23 studies investigated here, 14 reported gender with 10 providing data from female participants. In these studies, female participation was less than half that of males (32:72). Further research is warranted to investigate the patterns and forms of male and female processes of creative collaboration, collaborative creativity, and collaborative creative learning.

Through this systematic literature review of creative collaboration and collaborative creativity in the music laboratories of performance, improvisation and composition we have sought to interrogate the ways in which these concepts have been theorized and implemented. Whilst collaboration might be a long-standing practice in music (Delalande, 2016) it has a much shorter history as a research phenomenon and holds great potential for further investigation.

## Data Availability Statement

The original contributions presented in the study are included in the article/supplementary material, further inquiries can be directed to the corresponding author.

## Author Contributions

All authors listed have made a substantial, direct and intellectual contribution to the work, and approved it for publication.

## Conflict of Interest

The authors declare that the research was conducted in the absence of any commercial or financial relationships that could be construed as a potential conflict of interest.

## Publisher's Note

All claims expressed in this article are solely those of the authors and do not necessarily represent those of their affiliated organizations, or those of the publisher, the editors and the reviewers. Any product that may be evaluated in this article, or claim that may be made by its manufacturer, is not guaranteed or endorsed by the publisher.
